# Assessment of NO_2_ and SO_2_ emissions in the Tula industrial complex, Hidalgo, using the mobile mini-DOAS technique combined with the AERMOD dispersion model

**DOI:** 10.1007/s10661-025-14265-2

**Published:** 2025-07-05

**Authors:** Rosa Amalia González Rivero, Claudia Inés Rivera Cárdenas, Hugo Alberto Barrera Huertas, Miroslava Trueba Vázquez

**Affiliations:** 1 Faculty of Chemistry, Universidad Nacional Autónoma de México, Ciudad de Mexico, Mexico; 2 Instituto de Ciencias de La Atmósfera y Cambio Climático, Universidad Nacional Autónoma de México, Ciudad de Mexico, Mexico; 3 Laboratorio de Calidad del Aire y Sistemas de Información Geográfica, Instituto Politécnico Nacional, Ciudad de Mexico, Mexico; 4 Centro Mario Molina Para Estudios Estratégicos Sobre Energía y Medio Ambiente, Ciudad de Mexico, Mexico; 5 Escuela Nacional de Ciencias Biológicas, Instituto Politécnico Nacional, Ciudad de Mexico, Mexico

**Keywords:** Emissions, Nitrogen dioxide, Sulfur dioxide, Mini-DOAS, AERMOD

## Abstract

The Tula industrial complex, located in Hidalgo, is a major contributor to air pollution in Mexico, particularly NO_2_ and SO_2_. This study used the mobile mini-DOAS technique to quantify the emissions of these gases over a period of seven measurement campaigns conducted from 2022 to 2024. The measurement campaigns carried out in summer and winter recorded the highest emission fluxes, which correlated with a higher electricity demand. The variations observed were associated with fluctuations in industrial activity and meteorological conditions. The results for 2022 and 2023 showed a decrease in emissions compared to previous studies, which could be attributed to the recent introduction of natural gas for electricity generation. However, a significant increase was observed in 2024, which was correlated with an increase in activity at the Miguel Hidalgo Refinery. The emissions quantified with DOAS were used to implement the AERMOD model, which allowed the dispersion of NO_2_ and SO_2_ to be assessed. This model was implemented in a 15 km radius around the Tula industrial complex, considering meteorological and topographical variations in the region. The dispersion of pollutants was mainly directed to the west and south of the industrial complex. The results indicate that the concentrations of NO_2_ and SO_2_ exceeded the permissible limits established by official Mexican standards in several of the measurement campaigns. While all of the identified communities were affected by the emissions, Bomintzha, La Amistad, and San Miguel Vindho were the most affected due to their proximity to the complex, altitude, and prevailing wind direction.

## Introduction

Electric power generation and the petrochemical industry have been identified as significant contributors to air pollution (Liu et al., 2024; Rey et al., 2023). The main pollutants emitted into the atmosphere as a result of these activities are carbon monoxide (CO), nitrogen oxides (NOx), sulfur dioxide (SO_2_), particulate matter (PM), and carbon dioxide (CO_2_), a greenhouse gas. The impact of these atmospheric pollutants on human health and climate change has been the subject of many investigations (González et al., 2023; Keswani et al., 2022; Krastev et al., 2023; Manisalidis et al., 2020; Vandyck et al., 2022). Numerous studies around the world have demonstrated a correlation between exposure to these pollutants and an increase in the prevalence of respiratory diseases (Alejo et al., 2013; B. Cheng et al., 2024; Makgalemane et al., 2024). This phenomenon is particularly pronounced in urban areas with high levels of industrial pollution, such as Tula de Allende, Mexico.

Tula de Allende is a Mexican city located in the Hidalgo State, with a population of approximately 115,107 inhabitants (Gobierno de México, 2020). This city is home to one of the most important industrial complexes in Mexico. The Tula industrial complex consists of two thermoelectric plants, Francisco Pérez Ríos (FPRTP) and Ciclo Combinado (CCTP), which are operated by the Comisión Federal de Electricidad (CFE). They are considered the second largest generating thermoelectric plants in the country. The Miguel Hidalgo Refinery (MHR), owned by Petróleos Mexicanos (PEMEX), is also located there. In 2022 and 2023, it was considered the second most productive refinery of the national refining system of the United Mexican States. However, in 2024, it was the leading producer of PEMEX refined products. It has been estimated that the emissions from these entities contribute almost 97% of SO_2_ and 80% of NOx to the total emissions in the Hidalgo State and are responsible for 33% of pollution events in the Mexico City Metropolitan Area (MCMA) (Almanza et al., 2012, 2014; SEMARNAT & INECC, 2020). On the other hand, the Programa Institucional de Desarrollo de Servicios de Salud de Hidalgo 2023–2028 asserts that acute respiratory infections were the primary cause of disease in the Tula population in 2022 and 2023, and were not attributable to the COVID-19 pandemic (Secretaría de Salud, 2024). According to the national inventory of emissions of criteria pollutants, published by the Secretaría de Medio Ambiente y Recursos Naturales (SEMARNAT) in 2018, NOx and SO_2_ were the pollutants with the highest percentage of emissions from stationary sources in Hidalgo (SEMARNAT, 2024). This indicates that these pollutants are a primary concern in the region.

Several studies about atmospheric pollution have been performed in Tula. As part of the MILAGRO field campaign conducted in 2006, the differential optical absorption spectroscopy (DOAS) technique (Platt & Stutz, 2008) and the weather research and forecast model were used to verify if the emissions from the industrial complex affected air quality in the MCMA (Rivera et al., 2009b). In 2007, several authors analyzed the elemental composition of PM_10_ by particle-induced X-ray emission, the most abundant element being sulfur (Martínez et al., 2010). Other research demonstrated the contribution of PM emissions to local and regional air quality in the Tula industrial zone (Sosa et al., 2013). The influence of the Tula industrial complex on the MCMA was also demonstrated in 2014, this time analyzing NOx, SO_2_, and volatile organic compounds emissions using the multiscale climate chemistry model (García et al., 2014). In an experiment conducted at the industrial complex in 2017, emissions of 362.08 ± 300.14 ton day^−1^ of SO_2_ and 24.76 ± 12.82 ton day^−1^ of NO_2_ were obtained (Rivera & Arellano, 2024). The most recent studies conducted in Tula have been focused on proposing solutions to reduce gas emissions. The primary solution that has been proposed is to transition from fuel oil to natural gas exclusively (Sosa et al., 2020). The implementation of this initiative has been gradual since 2020, and its effectiveness remains to be ascertained. Despite efforts to reduce these emissions, pollution levels remain alarming. However, recent research has placed greater emphasis on evaluating the influence of the industrial complex on the MCMA, thereby leaving a gap in the evaluation of direct impacts on the communities in proximity to the complex. The limited coverage of local monitoring stations, coupled with the absence of real-time data, poses significant challenges in the accurate assessment of pollution levels in the area.

The passive DOAS technique has proven to be an effective tool for measuring the fluxes of trace gas emissions into the atmosphere. A major advantage of these instruments is their ability to perform real-time measurements. Consequently, numerous researchers have used this technique to study emissions from stationary sources such as volcanoes (Galle et al., 2002; Grutter et al., 2008; Johansson et al., 2009a, 2009b; Rodríguez & Nadeau, 2015; Bredemeyer et al., 2018; Lamotte et al., 2023) and industries (Johansson et al., 2009b; Rivera et al., 2009a, 2013, 2024; Zen et al., 2023). DOAS has also been used for the study of air quality degradation due to transportation (Huang et al., 2020; Ionov et al., 2022; Viatte et al., 2021). The integration of real measurements, such as those obtained with DOAS, with atmospheric dispersion models, such as AERMOD, is emerging as a robust strategy for the analysis and assessment of gaseous emissions from the Tula industrial complex. AERMOD is a steady-state Gaussian plume dispersion model recommended by the United States Environmental Protection Agency (EPA) (EPA, 2024). This model incorporates meteorological variables and geographic features. It simulates the distribution and transport of air pollutants. This model has been employed in some research to assess the dispersion of pollutants at refineries and thermoelectric plants (Ahmed & Jaf, 2022; Eslami et al., 2023, 2024; Mousavi et al., 2021; Siahpour et al., 2022). These previous studies have estimated emissions using calculations that assume a constant rate. The DOAS technique measures emissions directly in the area of interest, providing more accurate data. This study proposes to implement the results obtained with DOAS into the AERMOD dispersion model. Furthermore, the model has the capacity to generate relevant data to identify receptors in the vicinity of the complex that could be affected by NO_2_ and SO_2_ emissions. The integration of both tools is expected to provide a more realistic behavior of the dispersion of NO_2_ and SO_2_ emitted by the Tula industrial complex.

## Materials and methods

### Description of the study area

Tula de Allende has a territorial extension of 306 km^2^. The topography of the municipality is predominantly semi-flat, with plains and low hills. The city has a semi-arid climate, with hot summers and cool, dry winters. Rainfall is moderate and concentrated in the months of June to September. The rest of the year is mostly dry. The highest temperatures are recorded between March and May, while January is the coldest month. The relative humidity is generally low. The predominant wind directions are from the south from March to October, from the west in February and March, from the north in May, and from the east in June and part of December (Diebel & Norda, 2024).

**Fig. 1:**
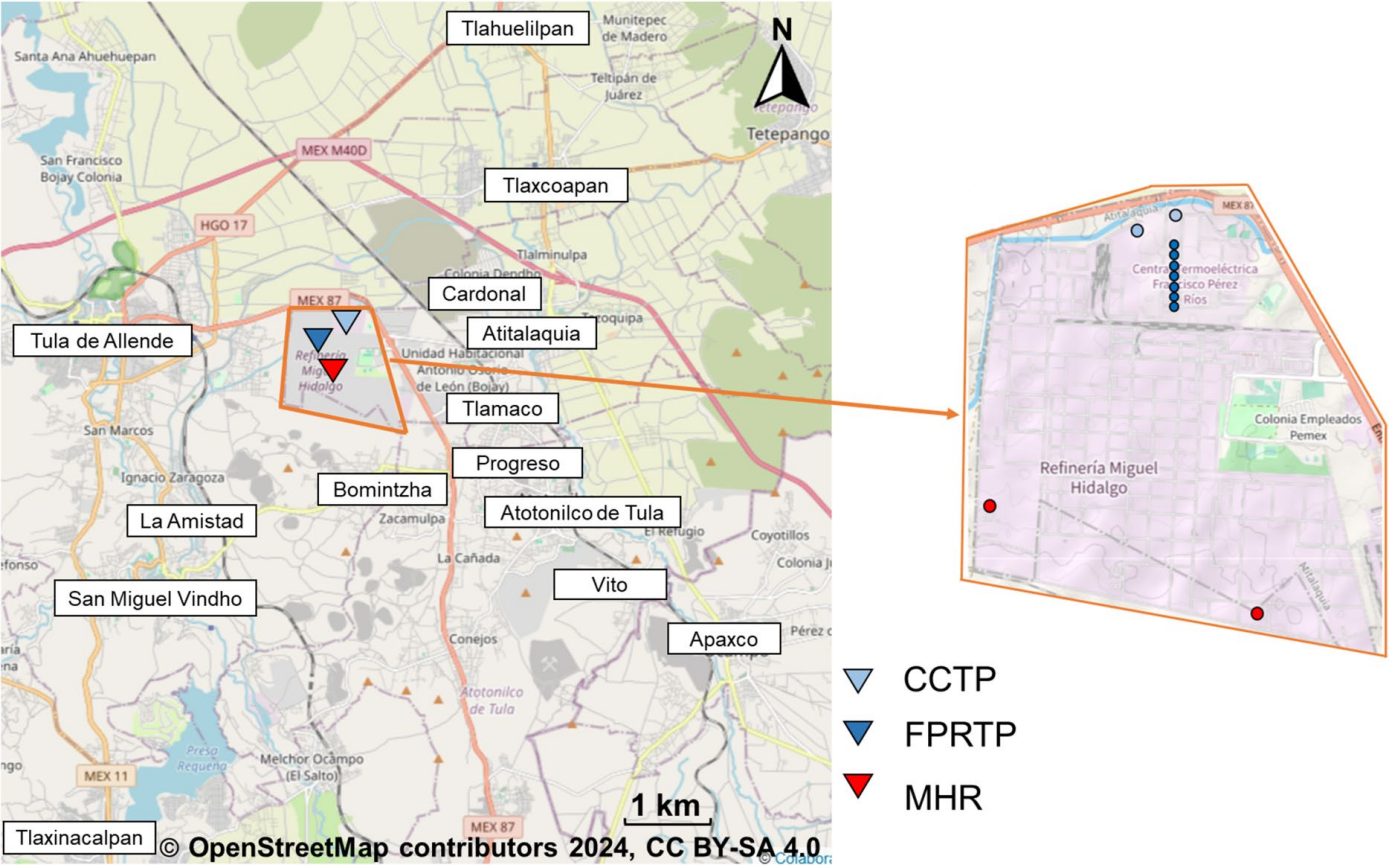
Map showing the location of the industrial complex, region outlined in brown. The light blue and dark blue triangles show the location of the CCTP and FPRTP, respectively. The red triangle shows the location of the MHR. The location of the communities that could be affected by pollutant emissions is also shown. The light blue points in the image on the right show the location of the CCTP emission sources. The dark blue points show the location of the FPRTP emission sources. The red points show the location of the MHR emission sources

The study of NO_2_ and SO_2_ emissions was conducted around the industrial complex, which is located approximately 7 km from the center of the city of Tula. The location of the FPRTP, CCTP, and MHR is shown in Fig. 1. The FPRTP has an installed capacity of 1605.60 MW and uses fuel oil. The CCTP uses natural gas and has a total generating capacity of 566.69 MW (Iniciativa Climática de México, 2024). The CCTP, by using the combined cycle of gas and vapor to generate electricity, produces fewer emissions compared to the FPRTP, which uses the traditional method of burning fossil fuels. The MHR has a crude oil processing capacity of 315,000 barrels per day (BPD). The facility operated at 45% of its maximum capacity in 2022 and 2023, increasing to 78.3% in 2024. The oil refining process mainly uses heavy crude oil, which contains between 2 and 4% sulfur by weight.

Table 1 presents a comprehensive set of data concerning communities in closest proximity to the industrial complex. The parameters considered in this study include the distance from the complex, the geographical location, the altitude of the communities, and the population of these communities. This information is critical for comprehending the potential repercussions of emissions on these communities.

**Table 1:** Characteristics of communities near the industrial complex

Locality	Distance to the industrial complex (km)	Coordinates	Altitude (m)	Inhabitants
Bomintzha	3.3	20°01′09.1″N–99°16′34.2″W	2234	4004
Progreso	4.3	20°01′12.8″N–99°14′44.8″W	2148	3310
Cardonal	5.2	20°03′44.1″N–99°14′15.5″W	2099	19,431
Tlamaco	5.2	20°02′05.7″N–99°13′44.6″W	2132	3885
La Amistad	5.4	20°00′25.7″N–99°19′11.3″W	2093	1681
Atitalaquia	6.5	20°03′36.4″N–99°13′17.0″W	2112	31,525
San Miguel Vindho	6.6	19°59′35.3″N–99°19′12.2″W	2134	8008
Tula de Allende	6.7	20°03′27.7″N–99°20′35.6″W	2032	115,107
Atotonilco de Tula	7.2	20°00′19.8″N–99°13′10.3″W	2165	62,470
Tlaxcoapan	7.7	20°05′25.4″N–99°13′22.6″W	2071	28,626
Vito	10.1	19°59′18.3″N–99°11′55.3″W	2164	4267
Tlahuelilpan	11.6	20°08′02.9″N–99°13′38.5″W	2152	19,067
Apaxco	13.1	19°58′27.2″N–99°10′27.8″W	2195	31,898
Tlaxinacalpan	14.5	19°55′23.2″N–99°20′28.5″W	2086	6490

### Mobile mini-DOAS instruments to quantify NO_2_ and SO_2_ emissions

The DOAS technique is widely used for gas monitoring, as it is based on the absorption of light by gaseous molecules. Depending on the light source, DOAS can be classified into active or passive techniques. Active DOAS uses artificial light sources, while passive DOAS uses natural light from the sun, moon, or stars. In this study, passive DOAS was used, specifically the scattered UV–VIS sunlight. Since light passes through the entire vertical extent of the atmosphere, a direct conversion of absorptions into concentrations is not possible (Galle et al., 2002; Platt & Stutz, 2008). The direct result of the DOAS observations corresponds to the differential slant column densities (DSCDs). The measurements were performed in a zenith-sky configuration (with the telescope pointing vertically), and in short periods of time (a few minutes). In addition, the trace gas layer is close to the ground, e.g., below the planetary boundary layer. For these reasons, the DSCDs are considered equal to differential vertical column densities (DVCDs) (S. Cheng et al., 2023a, 2023b; S. Cheng et al., 2023a, 2023b; Ma et al., 2013). As it was defined on Platt and Stutz (2008), a “differential” absorption is defined as the difference between the absorptions at two different wavelengths. This principle was used by Dobson in the 1930 s to determine the total column of atmospheric ozone by comparing the intensity of direct solar light of two wavelengths (Dobson & Harrison, 1926).

**Fig. 2:**
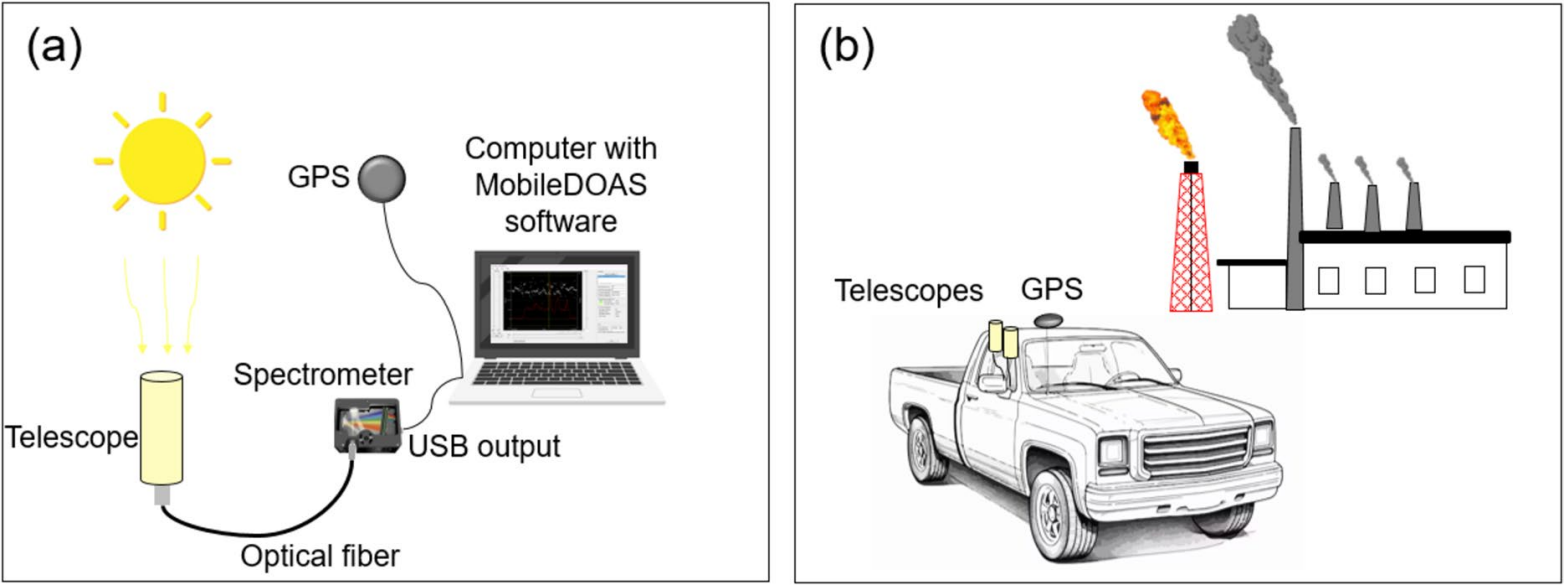
Images showing the DOAS measurement technique to quantify NO_2_ and SO_2_ emissions at the Tula industrial complex. **a** Schematic configuration of the mini-DOAS instrument. **b** Installation of mini-DOAS instruments in a car. The telescopes were placed on a window; the GPS devices on the roof and the rest of the components were placed inside the car

In this study, two calibrated mobile mini-DOAS instruments were used together to measure NO_2_ and SO_2_ emissions. The mini-DOAS instrument has several components (the schematic representation is shown in Fig. 2a). The first component is a receiving telescope with a single quartz lens, which has a focal length of 75 mm. The function of this component is to collect the zenith scattered light in the UV or VIS region. The field of view of the telescopes is 0.45°. To quantify SO_2_ in the UV range, we use a telescope with a Hoya U330 band-pass filter. This filter blocks visible light with wavelengths longer than 360 nm, maximizing the UV detection range. In contrast, to quantify NO_2_ in the VIS range, a telescope without a filter was used. A quartz optical fiber of 2 m length and an internal diameter of 0.8 μm (second component) is used to transfer scattered solar light between the telescope and a spectrometer. The spectrometer is the third component of the mini-DOAS system. This device divides the light into its wavelengths using an optical grating, which can be rotated to detect the desired wavelength ranges with high precision. The spectrometers used in our study are Ocean Optics, model USB2000 +, with a spectral resolution of 0.6 nm. The UV spectrometer, which was used to analyze the SO_2_ gas, operates within a spectral range of 273 to 432 nm. The visible spectrometer, which was used to analyze the NO_2_ gas, has a spectral range of 356 to 510 nm. The spectrometer contains a charge-coupled device (CCD) detector on which the spectrum is recorded. Finally, the spectrometer is connected to a computer (fourth component), where the MobileDOAS software (Zhang et al., 2021) was installed. The software takes spectra and evaluates the DVCDs of the analyzed gases in real time. A GlobalSat World Corporation GPS device, model BU-353S4, was used to georeference and record the time of each spectrum measured. This device was connected to the computer. These instruments were installed in a car (see Fig. 2b), which was used to drive transects around the industrial complex at a constant speed. The transects were designed based on the proximity to the main emission sources of the industrial complex and the predominant wind directions, in order to effectively capture the pollutant plumes. The transects were conducted between 8:00 and 17:00 h (local time). At the beginning of each transect, a background reference spectrum was recorded before passing through the plume (the spectrum should not contain significant levels of the pollutants). This was followed by a spectrum without any light (dark spectrum), which was measured by blocking the entrance of sunlight to the telescope. The integrated concentration of the target gas measured along the light path corresponds to the DVCD with respect to the background reference spectrum and the subsequently measured spectra. Spectra were recorded every 10 s (an average of about 180 to 360 spectra per measurement route, depending on how long the transect took).

Table 2 presents the schedule of the seven field measurement campaigns that were carried out. The NO_2_ and SO_2_ emissions were measured over a period of 3 years (2022, 2023, and 2024). This selection is intended to evaluate possible variations in emissions associated with changes in industrial activity. The months selected include periods of high solar radiation and high temperatures, such as March, August, and September. Measurements in months such as January, October, and November capture conditions of lower temperature and weak winds. This allows us to observe how emissions behave under high or low dispersion capacity.

**Table 2:** Schedule of measurement campaigns carried out in the Tula industrial complex

Measurement campaign number	Year	Month	Days (from-to)
1	2022	August, September	31, 1, 2
2	2022	November	16–18
3	2023	January	10–12
4	2023	March	21, 22
5	2023	October	21, 22
6	2024	January	22–27
7	2024	September	26–28

The evaluation of the spectra recorded in each measurement was performed using the QDOAS (version 3.2) (Danckaert et al., 2017) and MobileDOAS (version 6.3.1) (Zhang et al., 2021) software. In order to evaluate the spectra using the QDOAS software, the NO_2_ retrieval was performed in the wavelength range of 405 to 465 nm, while for SO_2_, it was performed from 307 to 317 nm. The result of a transect is the total number of molecules in a cross section of the plume. The retrieval process in the software is based on the fitting of measured spectra using a least square fitting procedure. The spectral structures included in the evaluation were NO_2_, SO_2_, ozone (O_3_), tetraoxygen (O_4_), water vapor, and a ring spectrum, which are fitted to resolve the contributions corresponding to the measured optical density. The cross sections for each gas were obtained from the literature (Bogumil et al., 2003; Hermans et al., 2003; Rothman et al., 2010; Vandaele et al., 1998, 2009). The MobileDOAS software uses the DVCDs expressed in parts per million per meter (ppmm) to calculate gas emission fluxes. This unit represents the amount of gas present along a gas column. Therefore, the DVCDs originally expressed in molecules cm^−2^ were converted to ppmm, assuming that 1 ppmm = 2.5 × 10^15^ molecules cm^−2^ (Kern et al., 2010). The mathematical expression used by the MobileDOAS software to calculate the flux (F) is described below (Eq. 1). The flux can be expressed in kg s^−1^ or ton day^−1^. The DVCD quantified from each spectrum is expressed in ppmm. The term “MCF” is a mass conversion factor, defined according to the molecular weight of the species of interest. The spatial distance between two sampling points is defined as “d”. The equation also includes the angle between the perpendicular travel direction and the wind direction. The wind speed at the height of the plume, expressed in m s^−1^, is defined as “WS”. Since the spectra of mobile DOAS measurements are discretized, the pollutant gases flux can be estimated by the summation along the measurement route.

1 $$\text{F}=\text{DVCD}\times \text{MCF}\times \left[\text{d}\times (\text{cos}\left(\text{travel angle}-\text{wind direction angle}+\frac{3\uppi }{2}\right))\right]\times \text{WS}$$

The information regarding wind speed and direction was obtained from the Real-time Environmental Applications and Display System (READY) webpage (NOAA, 2022; Rolph et al., 2017). The “sounding” option was selected, with the North American Mesoscale Forecast System (NAM) chosen as the meteorological information (horizontal resolution of 12 km and temporal resolution of 3 h). The day and time at which each measurement was taken was also specified. In order to obtain the vertical sounding of wind speed and direction, it was necessary to enter the site coordinates into the model (in our case we used latitude 20.058459°N, longitude 99.273792°W, which corresponded to a midpoint between the thermoelectric plants and the oil refinery). The webpage provided information about temperature, relative humidity, wind direction, and wind speed at different heights. To select the indicated meteorological data, it was necessary to calculate the effective plume height. This parameter was calculated following the procedure described by Gary A. Briggs (Briggs, 1982). The effective height is estimated as a function of unstable or neutral, or stable, atmospheric conditions. Different sets of equations are used for these conditions, as described by the author. Factors such as stack gas temperature, ambient temperature, stack exit gas velocity, stack diameter, and stack height have an influence on the plume rise.

### Assessment of emissions with the AERMOD model

AERMOD is a dispersion modelling tool that has been recommended by the EPA for the purpose of predicting the impacts of emission sources. The AERMOD model (version 12.0.0) uses two input data preprocessors: AERMET and AERMAP. The primary function of AERMET is the processing of meteorological data, integrating air dispersion concepts based on the structure and scaling principles of the planetary boundary layer turbulence (Seangkiatiyuth et al., 2011). The meteorological variables required on an hourly scale were temperature, relative humidity, wind speed, wind direction, and cloud cover. These dataset were obtained from the Meteoblue historical simulation archive (Meteoblue, 2024). AERMAP is a topographic data processor, which handles complex terrain using digital elevation data (DEM) provided by the United States Geological Survey (USGS). The primary function of this processor is to calculate the influence of the terrain on the dispersion. In addition, it is used to create the grid and the elevation of the receptors. The receptor grid used in this study consisted of 45 points. AERMOD has the capacity to account for turbulence caused by wind shear, as well as by buoyancy effects resulting from solar heating during the day and radiation cooling at night. In order to accurately account for these effects, AERMOD requires three land use parameters: albedo, Bowen ratio, and surface roughness. The albedo is defined as the fraction of total incident solar radiation reflected by the surface. The Bowen ratio is defined as the ratio of the sensible heat flux to the latent (evaporative) heat flux. The surface roughness length is related to the height of obstacles to the wind flow and is, in principle, the height at which the mean horizontal wind speed is zero (Burakowski et al., 2018). In the model, the albedo value introduced was 0.2075, the Bowen ratio was 1.625, and the surface roughness was 1.000. These values correspond to the typical characteristics of urban areas. The selected modelling area covered a radius of 15 km around the emitting sources to analyze some communities. Their descriptions can be found in Table 1.

The geographical coordinates and physical characteristics of the emission sources that had previously been identified in the industrial complex were also defined (see Fig. 1). The NO_2_ and SO_2_ emission sources identified were waste gas burners and stacks. The operational parameters of the emission sources were collected from the National Transparency Platform, a tool for consulting and requesting information on the production of public institutions in Mexico. In this study, the data were consulted through the requests identified with the numbers 1816400031520 and 1816400250121, facilitating their follow-up and verification. The emission fluxes used in the model were those obtained using the mobile mini-DOAS instruments. Three measurement campaigns for each pollutant were selected to assess their dispersion: two corresponding to the events with the highest emissions and one with the lowest emission. The output options used were 1 h and annual. The dispersion of the estimated hourly average concentrations for each data period was obtained. Furthermore, annual average concentrations were estimated for each year analyzed in this study. Finally, the concentrations estimated by the model were compared with the maximum permissible limits established in the Mexican Official Standards. The modelling includes diurnal and nocturnal simulation. Since no flux measurements were made at night, the emissions used in the model correspond to the average of the emissions measured during the day. Therefore, it is assumed that nocturnal industrial activity is similar to diurnal activity, considering the operational continuity of the emission sources.

## Results and discussion

### Emission fluxes obtained with the mobile mini-DOAS instruments

**Fig. 3:**
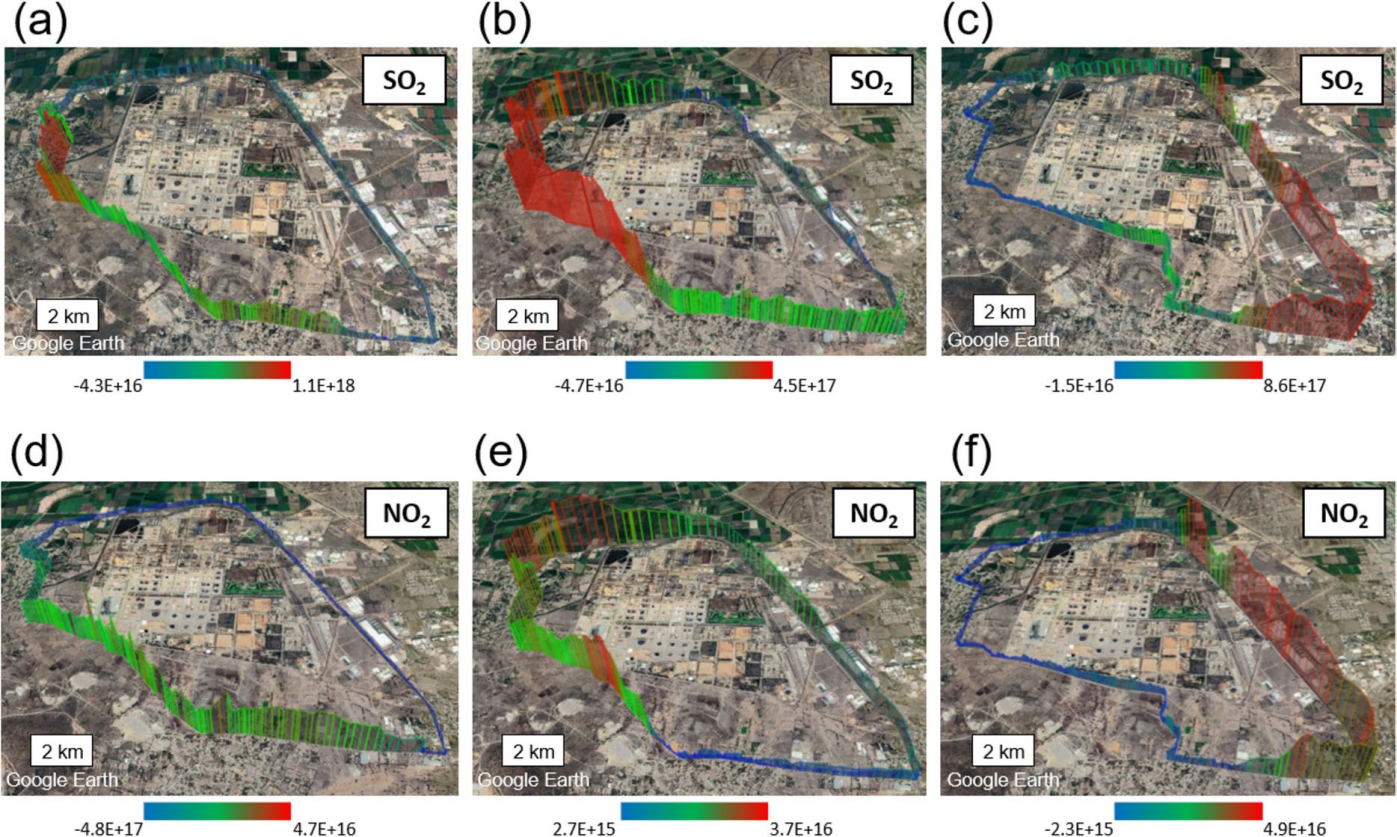
Examples of transects performed around the Tula industrial complex. The images show the spatial distribution of the DVCDs of SO_2_ and NO_2_ (expressed in molecules cm.^−2^). **a**, **d** Transect conducted on September 1, 2022, between 10:12 and 10:59. **b**, **e** Transect conducted on March 22, 2023, between 12:41 and 13:42. **c**, **f** Transect conducted on January 27, 2024, between 10:29 and 11:21

Three examples of transects conducted around the Tula industrial complex are presented in Fig. 3. These were obtained by exporting the results from the MobileDOAS software to Google Earth. The images show the spatial distribution of the DVCDs of SO_2_ and NO_2_, expressed in molecules cm^−2^. The colormap used to represent the spatial distribution uses the colors blue, green, and red to indicate increasing intensity. Figure 3a and d show the same transect where SO_2_ and NO_2_ measurements were performed on September 1, 2022, between 10:12 and 10:59 h. A clear orientation of a concentrated and little dispersed plume to the west of the industrial complex was observed in Fig. 3a. Figure 3d shows lower DVCDs and a more homogeneous distribution of NO_2_. The wind speed was 4.1 m s^−1^ and the solar radiation was moderate, which could indicate the presence of a slightly stable atmosphere during the measurement. The results of this transect were emission fluxes of 183.22 ton day^−1^ of SO_2_ and 19.59 ton day^−1^ of NO_2_. The transect conducted on March 22, 2023, between 12:41 and 13:42 h (see Fig. 3b and e), shows a plume orientation like that observed previously. However, the DVCDs of SO_2_ and NO_2_ appear to be more dispersed. This could be attributed to a wind speed of 5.3 m s^−1^ and atmospheric instability caused by solar heating at midday. This time, emission fluxes of 85.98 ton day^−1^ of SO_2_ and 3.75 ton day^−1^ of NO_2_ were obtained. Figure 3c and f show a transect performed on January 27, 2024, between 10:29 and 11:21 h. A wide and heterogeneous dispersion is observed for both pollutants, with the plume extending to the southeast of the industrial complex. This behavior could be related to the wind speed, which was 6.6 m s^−1^, moderate solar radiation, and fluctuations in wind direction during the measurement. This transect recorded emission fluxes of 393.26 ton day^−1^ of SO_2_ and 31.85 ton day^−1^ of NO_2_.

Table 3 shows a summary of the NO_2_ and SO_2_ emission fluxes obtained during the measurement campaigns. Also, the number of transects performed in each of them is shown, for a total of 125. As can be seen from the table, the NO_2_ emissions are significantly lower than SO_2_ emissions in all campaigns, which is consistent with the use of fossil fuels such as fuel oil generating more sulfur emissions. The standard deviations are particularly high in several campaigns, which indicates that emissions were not constant over the measurement transects. This can be attributed to variations in industrial activity, meteorological conditions (temperature, wind speed and direction, atmospheric stability), and the occurrence of unexpected events in the environment that may modify normal conditions such as fires, storms, thermal inversion, and the contribution of mobile sources in the area. In Hidalgo, winter is from December to part of March, while summer is from June to September. Measurement campaigns conducted in summer (1 and 7) and measurement campaign 6, conducted in winter, reported the highest SO_2_ emission fluxes. In measurement campaign 3, carried out in winter, a high value was also recorded. In these seasons, the demand for electricity increases because of the intensive use of cooling and heating systems. NO_2_ emissions reached their highest levels in measurement campaigns 6 and 7. In addition, high temperatures and increased solar radiation during summer contributed to photochemical reactions in the atmosphere, such as the oxidation of NO to NO_2_. For the rest of the measurement campaigns, the lowest emissions were recorded. This behavior could be associated with a higher operational activity of the CCTP in comparison to the FPRTP during the study periods. However, the hypothesis cannot be confirmed due to the absence of detailed information on the industrial operations of both plants.

**Table 3:** Summary of NO_2_ and SO_2_ emission fluxes obtained during the measurement campaigns

Number of measurement campaign	Number of transects	NO_2_ emissions (ton day-^1^)	Standard deviation (ton day-^1^)	SO_2_ emissions (ton day-^1^)	Standard deviation (ton day-^1^)
1	13	17.26	9.99	384.67	333.90
2	19	5.54	3.22	171.97	149.62
3	16	12.64	9.67	311.00	168.39
4	12	12.57	10.35	266.95	230.35
5	13	21.19	11.46	269.54	117.03
6	33	22.98	14.13	377.63	291.95
7	19	22.52	13.94	815.46	479.20

A variety of research estimates that the main errors influencing the calculation of emission fluxes when using a mobile mini-DOAS instrument are spectroscopic error, scattering error, wind field error, and drive speed (Lee et al., 2024; Rivera et al., 2009a; F. Wu et al., 2017; F. C. Wu et al., 2013). The first of these represents approximately 10%, and is attributable to temperature changes in the spectrometer, absorption cross-section error, spectral interferences, and fitting errors. The scattering error is highly dependent on weather conditions, including fog, rain, and cloud cover. Under ideal conditions (good visibility, blue sky or clouds clearly above the plume, and little or no condensation on the plume), an error of 10–30% is estimated. In moderate conditions (moderate to poor visibility, absence of transparency in the plume, and presence of clouds above the plume), a minimum error of 30% is estimated. In poor conditions (presence of low clouds or fog), the flux calculation in these conditions is not reliable. In our measurement campaigns, ideal or moderate conditions prevailed, estimating a scattering error of 10–30%. Wind field error refers to errors in wind speed and direction at the height of the plume. The overall error in the wind field is estimated to be 40% when balloon sounding information is not available, as in the case of our study. The accuracy of drive speed is about 1% resulting from GPS data. Finally, an overall error between 60 and 80% is estimated. However, a larger number of transects minimizes these sources of error, and the values obtained can be considered an accurate reflection of the real emission fluxes in the industrial complex.

An annual average of emissions was also estimated. Table 4 shows these values for the years 2022, 2023, and 2024. It also shows the annual gross generation data for both the CCTP and the FPRTP, as well as the crude oil processing by the MHR. The gross generation data of the CFE was obtained through the National Transparency Platform, with request number 330007724002293. The MHR data were obtained from PEMEX reports (Ayala, 2024; PEMEX, 2024). Emissions of both pollutants increased slightly from 2022 to 2023. During this period hydrocarbon processing at the MHR was constant. Therefore, the small variation observed could be related to a small increase in electricity generation at both thermoelectric plants. Emissions doubled in 2024, probably because of a 33% increase in the MHR activity. These data explain the high emissions obtained in the measurement campaigns carried out in 2024.

**Table 4:** Comparison of annual emission fluxes with electricity generation and crude oil processing data

Year	NO_2_ emissions (ton year^−1^)	SO_2_ emissions (ton year^−1^)	Gross generation CCTP (MWh)	Gross generation FPRTP (MWh)	Crude oil processing in MHR (BPD)
2022	3794.46	98,669.18	3,799,756.08	3,523,985.65	141,750
2023	5650.60	101,340.49	3,826,809.02	4,299,351.61	141,750
2024	7875.06	217,889.40	1,896,670.10^a^	2,473,309.83^a^	246,645

^a^The data concerning the gross generation of thermoelectric plants are until June 2024

#### Comparison with emission inventories and previous studies

Analyzing the data presented in Table 5, it can be seen that our results are analogous with the values reported in the literature. However, different methods are used in order to estimate emissions. The mini-DOAS instruments provide real-time measurements, while SEMARNAT calculates emissions using a process that combines reported data, directly monitored data, and estimates based on emission factors. Also, the spatial resolution of the data is not the same. While inventories cover the city of Tula or even the whole of the Hidalgo state, the measurements performed in this study were limited exclusively to the industrial complex. However, it is estimated that this complex is responsible for most of the emissions in the region. On the other hand, the differentiation between NOx, NO_2_, and NO is a very important point to analyze. While emission inventories report NOx emissions, the mini-DOAS instrument is designed to specifically measure NO_2_. In these industries, the emitted NO is initially oxidized to form NO_2_. This process is influenced by various atmospheric factors, including solar radiation, the presence of other pollutants, and ambient temperature. The rate and efficiency of this oxidation is not constant. Therefore, the amount of NO_2_ quantified by the mini-DOAS instrument does not necessarily represent the total NOx emitted. The discrepancies observed can be attributed to the fraction of NO that has not yet been converted to NO_2_ at the time of measurement. When comparing our results with the 2006 and 2017 studies, a decrease in emissions was observed in 2022 and 2023. This phenomenon could be associated with the implementation of combined cycle units, which optimize combustion efficiency and reduce harmful emissions significantly. However, a significant increase was observed in 2024, where the MHR activities prevailed.

**Table 5:** Literature data on NO_2_ and SO_2_ emission inventories

Year	Location	NO_2_ (ton year^−1^)	SO_2_ (ton year^−1^)	Method	Reference
2006	Tula industrial complex	8647.00	140,046.00	Mobile mini-DOAS	Rivera et al. (2009b)
2016	Total emissions in Tula (electric power generation)	3189.69^b^	109,237.26	Data combination	SEMARNAT (2024)
2017	Tula industrial complex	8766.00	140,256.00	Mobile mini-DOAS	Rivera and Arellano (2024)
2018	Total emissions in Hidalgo (electric power generation)	9205.45^b^	113,944.02	Data combination	SEMARNAT (2024)
	Total emissions in Hidalgo (oil and petrochemicals)	2769.20^b^	79,636.42		
2022	Tula industrial complex	3794.46	98,669.18	Mobile mini-DOAS	This work
2023	Tula industrial complex	5650.60	101,340.49		
2024	Tula industrial complex	7875.06	217,889.40		

^b^SEMARNAT emission inventories report NOx

### Results of the emission assessment using the AERMOD model

#### Hourly average concentrations of NO_2_ and SO_2_

**Fig. 4:**
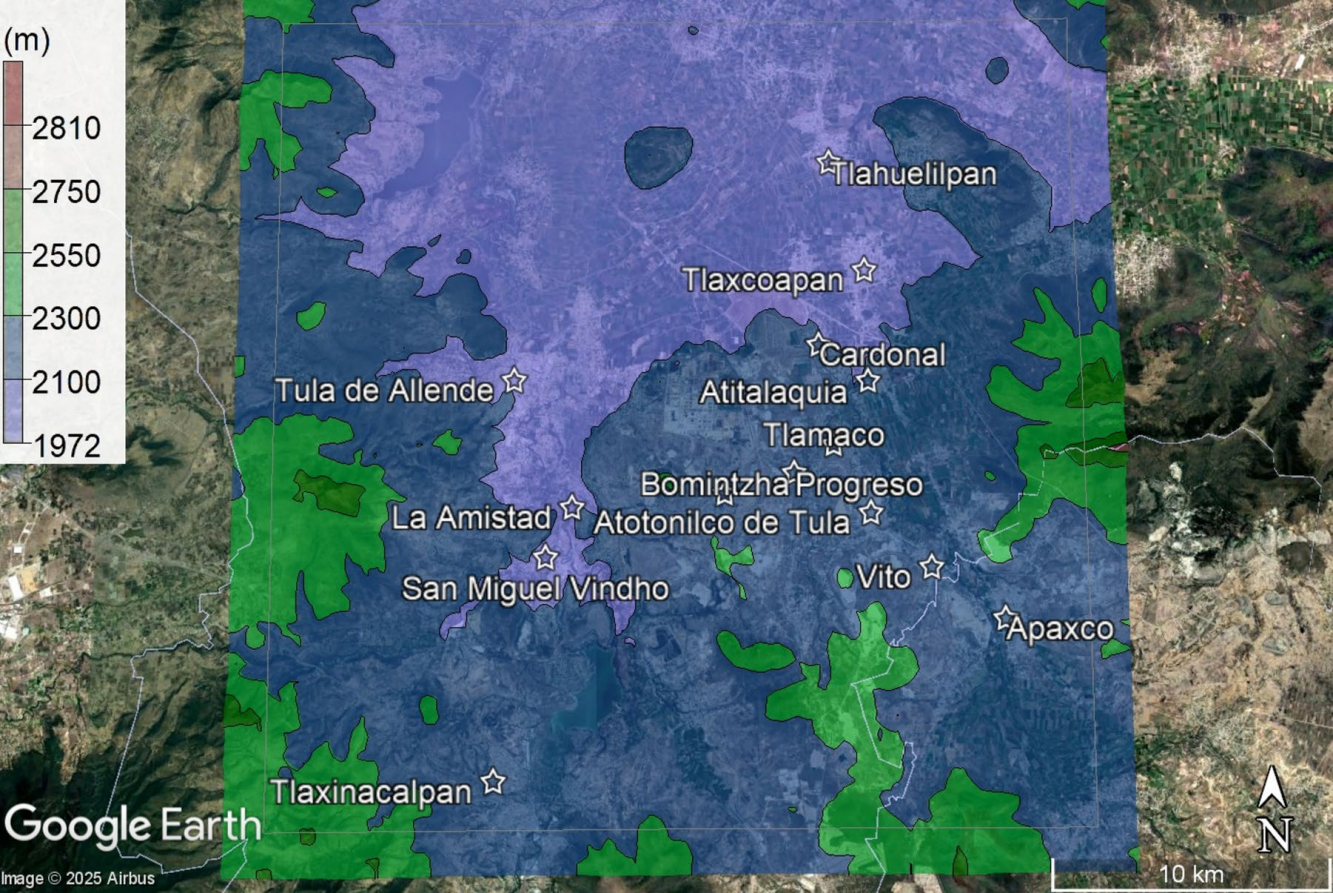
Topographical characteristics of the study area

The topography of the study area was determined using the AERMAP and Google Earth tools (see Fig. 4). The altitude of the study area varies between 1972 and 2810 m above sea level. The area is predominantly semi-flat, with the flattest communities being Tula de Allende and Tlaxcoapan. The highest community is Bomintzha.

**Fig. 5:**
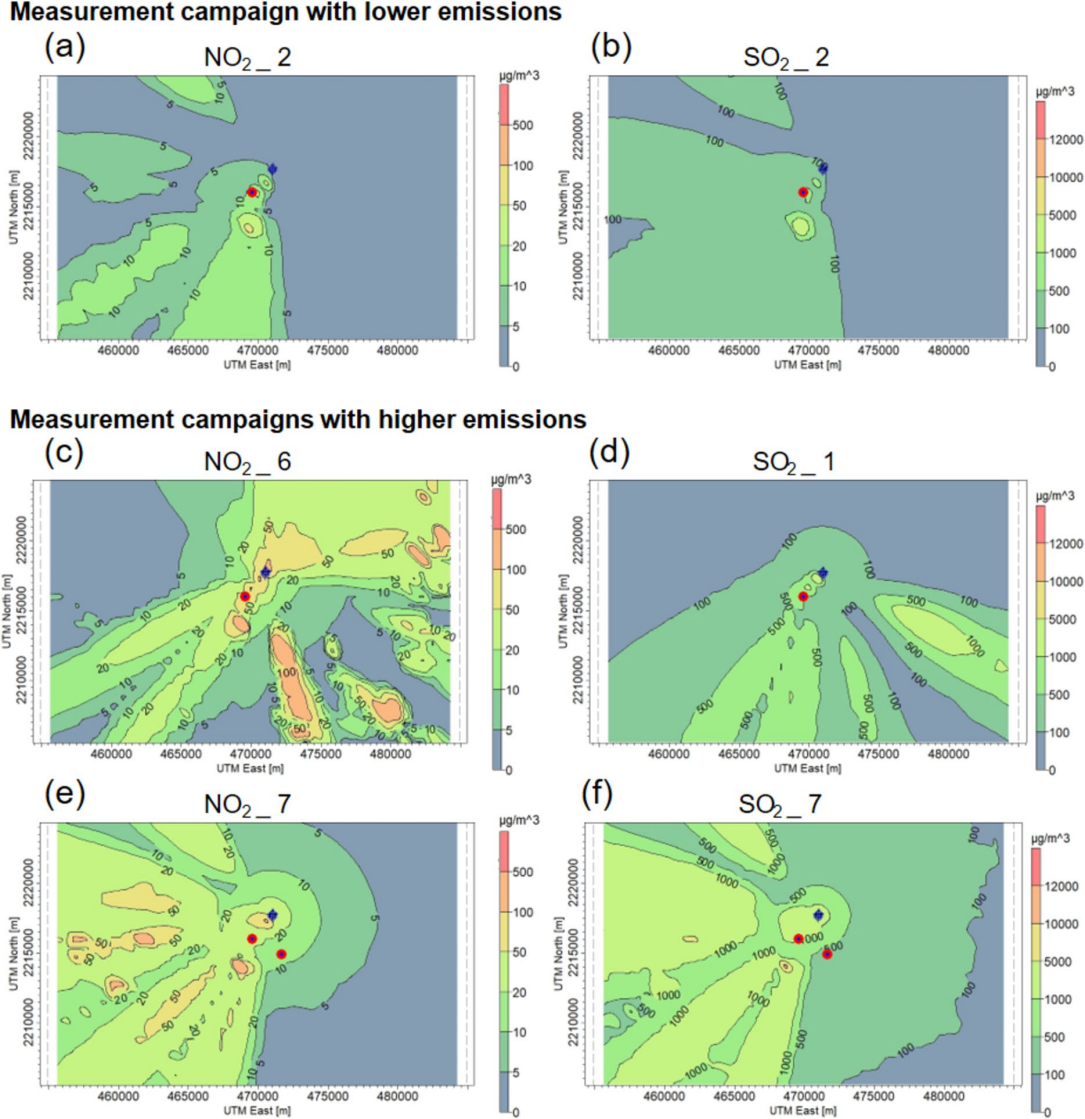
Dispersion maps of hourly average concentrations of NO_2_ and SO_2_, obtained using the AERMOD model, during the period of the measurement campaigns in which the lowest and highest emissions of both pollutants were obtained. **a**, **b** Dispersion of NO_2_ and SO_2_ concentrations in measurement campaign 2. **c** Dispersion of NO_2_ concentration in measurement campaign 6. **d** Dispersion of SO_2_ concentration in measurement campaign 1. **e**, **f** Dispersion of NO_2_ and SO_2_ concentrations in measurement campaign 7

Figure 5 shows the dispersion maps of the 1 h average for NO_2_ and SO_2_ concentrations obtained using the AERMOD model. In order to model the dispersion of NO_2_ concentrations, three measurement campaigns were selected: campaign 2 (Fig. 5a), which recorded the lowest emission, and campaigns 6 and 7 (Fig. 5c and e), which recorded the highest emissions. In the case of SO_2_, campaign 2 (Fig. 5b) was also identified as the lowest emission, while campaigns 1 and 7 (Fig. 5d and f) were selected due to their higher emissions of this pollutant. In this figure, areas of higher concentration are represented in orange and red, while lower concentrations are shown in blue. The location of the FPRTP stacks is denoted by blue cross markers. The stacks of the CCTP were not considered for modelling due to lack of knowledge of their operational parameters. The position of the MHR gas burners is indicated by the red circles.

Figure 5a and b show that both pollutants tend to concentrate mainly to the south and southwest of the industrial sources. In this direction, the communities of Bomintzha, La Amistad, Tula de Allende, and San Miguel Vindho are located, which were more affected by emissions. The highest concentrations were detected in an elevated area, reaching values of 51.7 µg m^−3^ for NO_2_ and 1455.1 µg m^−3^ for SO_2_. High concentrations were also recorded towards the northwest, a predominantly rural area. Figure 5c shows how NO_2_ tends to disperse in several directions but is mainly concentrated to the south-southeast of the emitting sources. Elevated concentrations are also observed to the east-northeast. The maximum concentration recorded was 470.3 µg m^−3^. In this case, Bomintzha was the area most affected by the emissions. Figure 5d shows that SO_2_ is dispersed towards the southeast and south-southwest. Some communities located in these directions are La Amistad, San Miguel Vindho, and Tlamaco. The highest concentration was recorded inside the industrial complex, with a value of 2050.0 µg m^−3^. Figure 5e and f show that both pollutants are dispersed in the same direction. The communities most affected were Bomintzha, La Amistad, Tula de Allende, and San Miguel Vindho. For both pollutants, the highest estimated concentration was recorded in the south-southwest of the industrial complex, in an elevated area (2350 m above sea level). In this area, 328.7 µg m^−3^ of NO_2_ and 10,801.8 µg m^−3^ of SO_2_ were recorded. It has been observed that SO_2_ tends to concentrate in the vicinity of emitting sources, while NO_2_ is distributed towards more distant areas. This trend was also observed in Fig. 5a and c. This behavior can be attributed to the higher density of SO_2_ (2.63 kg m^−3^) in comparison to NO_2_ (1.45 kg m^−3^). Therefore, NO_2_ is more easily transported in the atmosphere, particularly in windy or turbulent conditions. In the latest measurement campaign, a new emitting source was identified at the Tula industrial complex. This source was a gas burner from the MHR.

From the different maps in Fig. 5, we can see that there are differences in the dispersion patterns obtained. In some areas, uniform concentric dispersion suggests relatively stable meteorological conditions, while in others, dispersion in multiple directions may indicate changes in wind direction and speed. In addition, the modelling used considers the hourly variability of the wind, which contributes to the representation of these patterns in the dispersion maps.

Mexican Official Standards NOM-023-SSA1-2021 and NOM-022-SSA1-2019 define the criteria for assessing ambient air quality with respect to NO_2_ and SO_2_, respectively. These standards establish a maximum concentration of 200 µg m^−3^ of NO_2_ and 196.5 µg m^−3^ of SO_2_ for a 1-h exposure time. From the results of the model, it can be seen that the maximum concentration of NO_2_ recorded during measurement campaign 2 does not exceed the limit established by the standard. However, in measurement campaigns 6 and 7, the maximum concentrations exceed the permitted thresholds. On the other hand, in the case of SO_2_, the maximum concentrations recorded during the three campaigns exceed the limit established by the official Mexican standard. It is worth mentioning that not only the highest modelled concentration value exceeds the maximum permissible limit.

The highest concentrations of NO_2_ and SO_2_ were obtained during measurement campaigns 6 and 7, respectively. The lowest concentrations observed correspond to measurement campaign 2. These results demonstrate a high degree of correlation with those obtained using the mobile mini-DOAS instruments.

#### Assessment of NO_2_ and SO_2_ impact areas

Table 6 shows the maximum concentrations of NO_2_ and SO_2_ obtained by the AERMOD model in each location during the measurement campaigns analyzed. When no values are present, it means that the dispersion did not affect that community. Analyzing the table, we can see that the NO_2_ concentrations estimated by the AERMOD model for measurement campaign 2 show that no community exceeded the established limits. In this campaign, Tlaxinacalpan is the only community where the SO_2_ concentration is slightly higher than the standard. In measurement campaign 1, the communities with the highest SO_2_ concentrations were La Amistad, San Miguel Vindho, and Tlamaco, exceeding two to four times the established thresholds. Tlaxinacalpan was also a bit higher than the threshold. In measurement campaign 6, Bomintzha was the area most affected by NO_2_ emissions. Although it did not exceed the limit established by the standard, the estimated value was considerably close. In measurement campaign 7, no community recorded NO_2_ concentrations exceeding the standard. However, the estimated SO_2_ concentrations in Bomintzha, Progreso, and Cardonal exceeded the standard values slightly. In addition, La Amistad, Tula de Allende, and San Miguel Vindho recorded SO_2_ concentrations five times higher than the recommended concentration threshold.

**Table 6:** AERMOD-estimated NO_2_ and SO_2_ maximum concentrations in communities near the industrial complex

	**NO**_**2**_** (µg m**^**−3**^**)**			**SO**_**2**_** (µg m**^**−3**^**)**		
**Measurement campaign number**	**2**	**6**	**7**	**1**	**2**	**7**
Bomintzha	5.0	182.7	9.7	106.9	94.5	249.4
Progreso	1.2	5.8	8.5	70.4	24.1	252.7
Cardonal	1.3	36.6	7.9	71.7	27.1	251.5
Tlamaco	1.1	5.3	7.2	718.6	29.9	170.1
La Amistad	6.9	10.0	54.5	500.2	152.4	913.1
Atitalaquia	1.0	9.2	7.6	69.1	23.2	164.6
San Miguel Vindho	5.4	9.8	53.7	481.9	152.4	1224.5
Tula de Allende	3.8	-	45.8	72.0	119.4	1287.5
Atotonilco de Tula	0.9	-	5.3	114.0	21.1	132.9
Tlaxcoapan	1.0	29.8	5.7	49.1	23.7	126.2
Vito	-	-	-	115.3	-	-
Tlahuelilpan	-	5.0	4.5	35.9	-	123.5
Apaxco	-	-	-	115.3	-	-
Tlaxinacalpan	9.8	32.5	23.5	255.6	198.4	-

In general, the communities most affected were Bomintzha, La Amistad, and San Miguel Vindho, due to its proximity to the industrial complex and its location at one of the highest altitudes. They were also strongly influenced by wind direction. As a result, these communities recorded the highest NO_2_ and SO_2_ concentrations. Despite the sparse population density of these areas, a total of 13,693 inhabitants are acutely affected by the emissions from the Tula industrial complex. In contrast, the communities that experienced the least impact were Vito and Apaxco. This can be attributed to their distance from the industrial complex and situated in a different prevailing wind direction. In Tula de Allende, the direct influence of emissions is lower compared to other locations closer to the industrial complex. However, SO_2_ concentrations were estimated to exceed established thresholds. In contrast to other communities that have been more severely impacted, this city is the most populated of those studied, meaning that a significant proportion of the population is constantly exposed to elevated concentrations of NO_2_ and SO_2_. This chronic exposure can have a serious impact on the health of the population, especially in vulnerable groups.

Although not included in this study, the MCMA, located to the south of the complex, probably experiences a deterioration in air quality, a phenomenon that has been previously observed in other research.

#### Annual average concentrations of NO_2_ and SO_2_

**Fig. 6:**
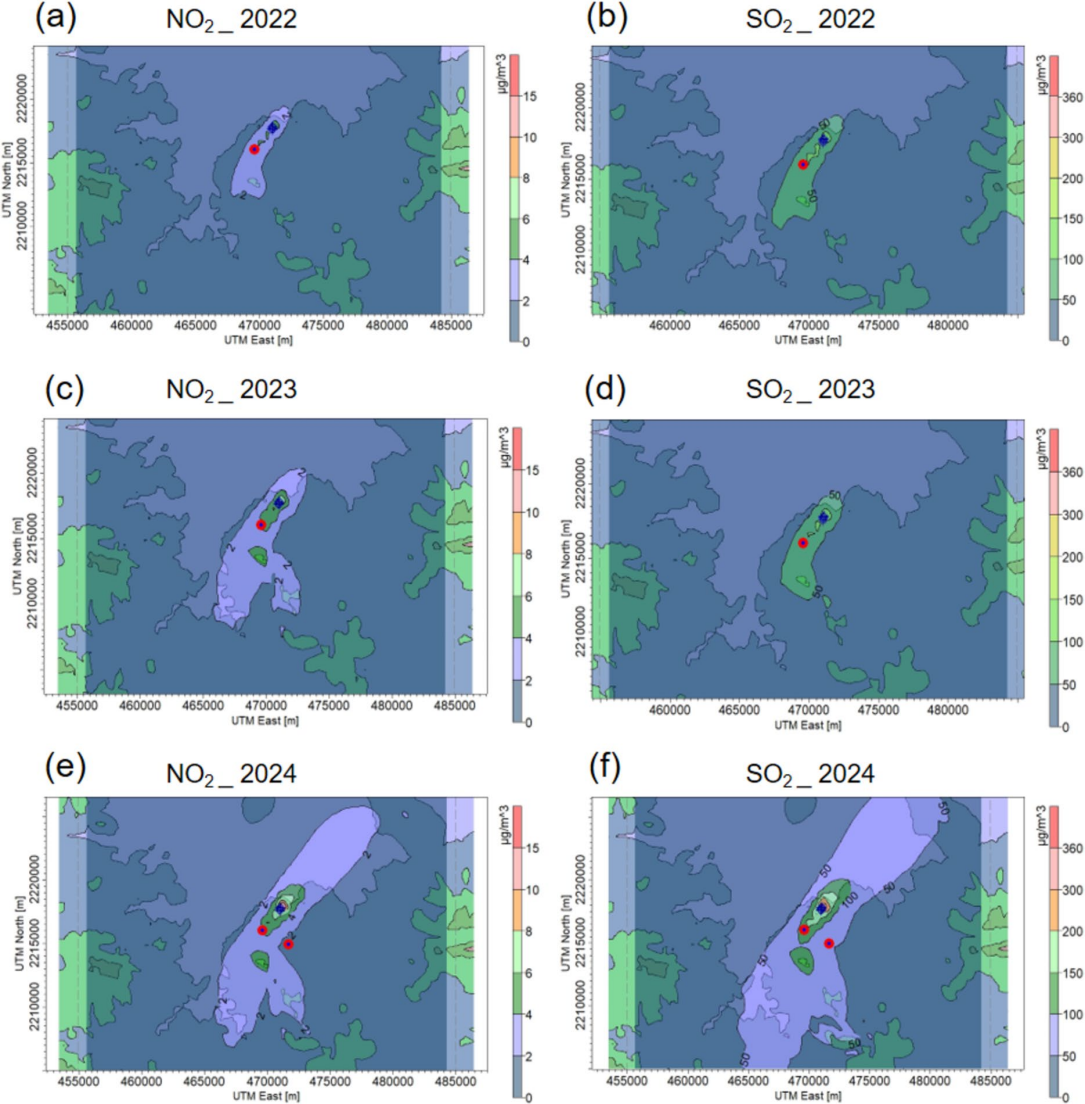
Annual dispersion of NO_2_ and SO_2_ in the study area obtained by the AERMOD model. **a**, **c**, **e** Dispersion of NO_2_ concentration during the years 2022, 2023, and 2024, respectively. **b**, **d**, **f** Dispersion of SO_2_ concentration during the years 2022, 2023, and 2024, respectively

Figure 6 shows the spatial distribution of the average annual concentration of NO_2_ and SO_2_ for the years 2022, 2023, and 2024. In this modelling exercise, the annual emissions presented in Table 4 were used. Figure 6a, c, and e represent the NO_2_ dispersion in the years 2022, 2023, and 2024, respectively. In these figures, the highest annual average NO_2_ concentrations are observed in the vicinity of the emitting sources. The dispersion follows a predominant pattern towards the southwest and south-southeast, suggesting an influence of the prevailing winds in the region. Although there are slight interannual variations, the overall distribution of NO_2_ remains similar, with maximum concentrations of 5.6 µg m^−3^ in 2022, 8.1 µg m^−3^ in 2023, and 12.9 µg m^−3^ in 2024. The annual concentration of NO_2_ is limited to 40 µg m^−3^ according to NOM-023-SSA1-2021; thus, the estimated levels are below the normative value. Figure 6b, d, and f represent the SO_2_ dispersion in the years 2022, 2023, and 2024, respectively. In these figures, the highest annual average SO_2_ concentrations are also found in the vicinity of the emitting sources, but with more extended exposure. The predominant wind patterns coincide with NO_2_. The interannual variation is highest in 2024. The estimated maximum concentrations were 144.9 µg m^−3^ in 2022, 146.0 µg m^−3^ in 2023, and 359.9 µg m^−3^ in 2024. Normative NOM-022-SSA1-2019 does not refer to any standard value in a year for the annual SO_2_ concentration. However, this value is limited to 66 µg m^−3^ according to NOM-022-SSA1-2010; thus, the estimated levels are above the permitted thresholds in the 3 years. The NO_2_ modelling in 2022 and 2023 indicates that the communities with the highest exposure to emissions were La Amistad, San Miguel Vindho, and Bomintzha. In 2024, the impact was extended to Tlaxcoapan and Tlahuelilpan. In the case of SO_2_, these five communities, together with Cardonal, were the most impacted during the 3 years of the study.

## Conclusions

In this study, the mobile mini-DOAS technique combined with the AERMOD dispersion model was used to assess NO_2_ and SO_2_ emissions from the Tula industrial complex and their impact on nearby communities. The emission fluxes obtained with the mini-DOAS technique showed significant variations between measurement campaigns. These variations were due to changes in industrial activity and meteorological conditions in the region. The highest emissions were recorded during the periods of highest energy demand. Although the use of natural gas has shown some potential to reduce emissions of NO_2_ and SO_2_, this strategy has not been sufficient at the Tula industrial complex. The observed variations in the hourly dispersion profiles indicate that meteorological conditions, in particular wind speed and direction, are crucial for the spatial distribution of pollutants in the atmosphere. The trajectory of the emitted air pollutants is also strongly influenced by the local topography. These conditions favored the dispersion of NO_2_ and SO_2_ mainly to the west and south of the industrial complex. On the other hand, the concentrations obtained with the AERMOD model exceeded the thresholds established by the Official Mexican Standards in almost all the measurement campaigns. This behavior was significant in communities such as Bomintzha, La Amistad, and San Miguel Vindho. On the other hand, Vito and Apaxco, far from the industrial complex and outside the main wind paths, were the least affected. In the annual study, the results show that, although NO_2_ concentrations remain within the regulatory limits, SO_2_ levels exceed the permitted values in all years analyzed, representing a significant environmental risk. The dispersion of both pollutants is influenced by prevailing winds, extending the geographical impact in 2024. The most affected communities were La Amistad, San Miguel Vindho, Bomintzha, Tlaxcoapan, Tlahuelilpan, and Cardonal. It can be concluded that the AERMOD model provides useful information for identifying vulnerable areas by incorporating real and variable values obtained with the mini-DOAS instruments. One of the main limitations of the model is that it does not take into account the chemical reactions of the pollutants, which could be addressed in future research through the use of more complex models. This work also highlights the need to implement more robust public policies. These include a full switch to natural gas, improved environmental regulation, and the expansion of monitoring networks.

## Data Availability

No datasets were generated or analysed during the current study.
